# Bio-behavioural HIV and STI surveillance among men who have sex with men in Europe: the Sialon II protocols

**DOI:** 10.1186/s12889-016-2783-9

**Published:** 2016-03-02

**Authors:** Lorenzo Gios, Massimo Mirandola, Igor Toskin, Ulrich Marcus, Sandra Dudareva-Vizule, Nigel Sherriff, Michele Breveglieri, Martina Furegato, Cinta Folch, Laia Ferrer, Alexandra Montoliu, Christiana Nöstlinger, Wim Vanden Berghe, Sharon Kühlmann-Berenzon, Inga Velicko, Sónia Dias, Barbara Suligoi, Vincenza Regine, Danica Stanekova, Magdalena Rosińska, Saulius Caplinskas, Irena Klavs, Ivailo Alexiev, Alexandru Rafila

**Affiliations:** Veneto Region - Department of Health, CReMPE - Regional Coordination Centre for European Project Management, the Verona University Hospital, Verona, Italy; Department of Pathology, Infectious Diseases Section, the Verona University Hospital - Veneto Region, Verona, Italy; Department of Reproductive Health & Research, World Health Organization, Geneva, Switzerland; Department for Infectious Diseases Epidemiology, Robert Koch-Institute, Berlin, Germany; Centre for Health Research, University of Brighton, Brighton, UK; Institut Catala d’Oncologia (ICO), Centre for Epidemiological Studies on HIV/STI in Catalonia (CEEISCAT), Agencia de Salut Publica de Catalunya (ASPC), Hospital Universitari Germans Trias i Pujol, Barcelona, Spain; Department of Public Health, Institute of Tropical Medicine, Antwerp, Belgium; Department of Monitoring and Evaluation, Public Health Agency of Sweden, Solna, Sweden; Institute of Hygiene and Tropical Medicine & GHTM, Universidade Nova de Lisboa, Lisbon, Portugal; Centro Operativo AIDS, Dipartimento di Malattie Infettive, Parassitarie ed Immunomediate, Istituto Superiore di Sanità, Rome, Italy; NRC for HIV/AIDS, Slovak Medical University, Bratislava, Slovak Republic; Department of Epidemiology, National Institute of Public Health, National Institute of Hygiene, Warsaw, Poland; Centre for Communicable Diseases and AIDS, Vilnius, Lithuania; National Institute of Public Health, Ljubljana, Slovenia; National Reference Laboratory of HIV, National Center of Infectious and Parasitic Diseases, Sofia, Bulgaria; National Institute of Infectious Diseases Prof. Dr. Matei Bals, Bucharest, Romania

**Keywords:** HIV, Surveillance, MSM, Time-location sampling, Respondent-driven sampling

## Abstract

**Background:**

Globally, the HIV epidemic continues to represent a pressing public health issue in Europe and elsewhere. There is an emerging and progressively urgent need to harmonise HIV and STI behavioural surveillance among MSM across European countries through the adoption of common indicators, as well as the development of trend analysis in order to monitor the HIV-STI epidemic over time. The Sialon II project protocols have been elaborated for the purpose of implementing a large-scale bio-behavioural survey among MSM in Europe in line with a Second Generation Surveillance System (SGSS) approach.

**Methods/Design:**

Sialon II is a multi-centre biological and behavioural cross-sectional survey carried out across 13 European countries (Belgium, Bulgaria, Germany, Italy, Lithuania, Poland, Portugal, Romania, Slovakia, Slovenia, Spain, Sweden, and the UK) in community settings. A total of 4,966 MSM were enrolled in the study (3,661 participants in the TLS survey, 1,305 participants in the RDS survey). Three distinct components are foreseen in the study protocols: first, a preliminary formative research in each participating country. Second, collection of primary data using two sampling methods designed specifically for ‘hard-to-reach’ populations, namely Time Location Sampling (TLS) and Respondent Driven Sampling (RDS). Third, implementation of a targeted HIV/STI prevention campaign in the broader context of the data collection.

**Discussion:**

Through the implementation of combined and targeted prevention complemented by meaningful surveillance among MSM, Sialon II represents a unique opportunity to pilot a bio-behavioural survey in community settings in line with the SGSS approach in a large number of EU countries. Data generated through this survey will not only provide a valuable snapshot of the HIV epidemic in MSM but will also offer an important trend analysis of the epidemiology of HIV and other STIs over time across Europe. Therefore, the Sialon II protocol and findings are likely to contribute significantly to increasing the comparability of data in EU countries through the use of common indicators and in contributing to the development of effective public health strategies and policies in areas of high need.

## Background

The HIV epidemic is still representing a pressing public health issue worldwide and in Europe as well [1]. Current documents published by the European Centre for Disease Control (ECDC) and focusing on the WHO European Region clarify that 136,235 new HIV diagnoses were reported in 2013, with a rate of 15.7 per 100,000. A total of 1,715,434 infections diagnosed in the WHO European Region represent the current number of persons leaving with HIV.

In terms of transmission, according to the 2013 data, the highest proportion of new HIV diagnoses was reported in men who have sex with men (MSM): this specific sub-population accounts for 42 % of all new infections in EU/EEA countries (compared to 40.4 % in 2012) [2].

For this specific population, in EU/EEA countries where data are available, since 2004 HIV diagnoses have increased by 33 %, confirming that MSM could be considered as a very high risk population for acquiring HIV, as a considerable part of the new HIV infected people across Europe is reported among this sub-population.

In addition, in younger MSM an increase of new diagnoses is dramatically reported for the EU/EEA area: between 2004 and 2013, the number of new HIV positive MSM aged 20-24 years almost doubled, whilst in adult MSM (30–39 years old) the estimates remain relatively stable. These high estimates (for the new diagnoses in particular) could be partially due to increasing HIV testing behaviours, but they could also indicate onward transmission in this specific population. Indeed, with regards the latter, according to the current scientific literature an increase of high risk sexual practices (such as unprotected anal intercourse) has been reported among MSM across Europe [1, 3]

In terms of serum-status awareness, almost one third of those infected in Europe are estimated to be unaware of their seropositive status [1].

Data seems to suggest that the current situation is becoming increasingly critical from a public health perspective.

Set within this context, HIV diagnosis has become a key surveillance activity for monitoring the HIV epidemic especially in ‘hard-to-reach’ populations such as MSM. Reliable data, including trends in risk behaviours over time, are of crucial importance in order to understand whether and by how much rates are increasing or decreasing and which (sub-) populations are affected the most. Consequently, the international literature, such as UNAIDS and WHO reports and publications [4], have stressed the need for public health to embrace three main approaches in monitoring and controlling the epidemic: (i) a structured surveillance system method; (ii) the use of common set of indicators adopted across Europe, and; (iii) specific prevention campaigns targeting MSM and testing promotion as the cornerstone of the HIV response.

In terms of structured surveillance initiatives and in response to the growing awareness of the urgency for a comprehensive and effective response, a number of European countries have implemented surveys specifically targeting MSM [5] focusing primarily on the monitoring of risk behaviours. However, a Second Generation Surveillance System (SGSS) approach [4], whereby both biological (e.g. oral fluid) and behavioural data (e.g. from questionnaires) are collected and analysed jointly, has only been adopted in a limited number of countries [6]. This is problematic and limits the value of such targeted initiatives due to, amongst other things, a lack of reliability and comparability of data at the European level [7]. A SGSS approach is defined by the WHO as the “regular, systematic collection, analysis and interpretation of information for use in tracking and describing changes in the HIV/AIDS epidemic over time” [8]. SGSS is important because it not only allows the public health sector to monitor the spread of infections in a given population and to analyse trends over time, but it can also facilitate countries to improve their planning and evaluation of prevention and treatment activities.

With regards to the second approach (i.e. the use of a common set of indicators adopted across Europe to monitoring and controlling the epidemic), despite some European countries adopting a SGSS approach, implementation is regrettably patchy and not systematic. Furthermore, even where examples of a SGSS approach are evident, there is often considerable variation between countries in terms of specific indicators utilised and reported. Such challenges highlight the need for HIV and STI behavioural surveillance among MSM across EU countries to be harmonised for high-risk population such as MSM [5]. This call to action has been taken up by the United Nations General Assembly in 2001, which in a Special Session on HIV/AIDS proposed the construction of a set of core indicators, namely the UNGASS indicators [9, 10] to kick-start such harmonisation in the collection of data at the international level. These UNGASS indicators have been revised and updated periodically until post-2012 when they became known as the Global AIDS Response Progress Reporting (GARPR) indicators (see [11] for the most recent guidelines, released at the beginning of 2014).

In terms of the third approach (i.e. targeted prevention campaigns), such specific HIV/STI prevention campaigns targeting MSM and promoting testing represent the cornerstone of the HIV response. Yet although many EU Member States (public health sectors and voluntary/third sectors) have routinely and for some time, implemented prevention programmes targeting MSM specifically, current prevention and treatment strategies appear to be not sufficient [12] . It has to be acknowledged that the evidence-base for primary HIV prevention does not reach the same degree of graded evidence as bio-medical prevention approaches due to the complexity of intertwined behavioural and structural factors. This is not surprising given that health promotion and public health interventions are complex, non-linear, and multi-layered processes often with no simple measurable outcomes. They are therefore difficult to evaluate, resulting in a general lack of evidence for which HIV programmes might be the most effective [13].

To-date, few studies have been able to address simultaneously all three of the approaches to tackling the HIV epidemic as advocated by UNAIDS and the WHO. However, of those studies that have embraced such an approach, community-based surveys targeting MSM that adopt bio-behavioural measures (e.g. oral fluid testing for HIV and behavioural questionnaires) have found high levels of HIV prevalence and risk behaviours (such as unprotected anal intercourse) as well as critical levels of HIV testing uptake considering the high frequency of unprotected anal intercourse with different partners [14, 15]. Such studies continue to highlight the urgent need for large-scale reliable and comparable second generation surveillance data on MSM that are paired with meaningful STI/HIV prevention, treatment and care.

Consequently, and in line with the most recent EC communication on combating HIV/AIDS in the European Union and neighbouring countries (2009–2013), the overall objective of the Sialon II project is to carry out and promote combined and targeted HIV/STI prevention for MSM complemented by second generation HIV/STI surveillance in collaboration with UNAIDS and WHO. The project is funded by the European Commission under the Second Programme of Community Action in the Field of Health (2008–2013). The Sialon II project is based on the experiences and lessons learned from the former Sialon project, funded under the 2003–2008 Public Health Programme (Work Plan 2007) [15].

In this paper, the Sialon II project protocols are presented which included three parts: (i) the formative research phase (including prevention needs assessment) with the objective to identify the specific community-based settings to carry out data collection and to assess local prevention needs; (ii) the use of innovative surveillance methodologies with the objective to access ‘hard-to-reach’ MSM in community-based settings; (iii) HIV (HIV1/2) and STI (Syphilis and Hepatitis [HBV/HBC]) testing algorithms, the implementation of targeted prevention activities with the objective to respond to immediate prevention needs when conducting the data collection.

## Methods

The Sialon II project is a multi-centre biological and behavioural cross-sectional survey carried out across 13 European countries including: Belgium, Bulgaria, Germany, Italy, Lithuania, Poland, Portugal, Romania, Slovakia, Slovenia, Spain, Sweden, and the UK. The survey has been implemented using the same methodologies (protocols, UNGASS/GARPR indicators, epidemiological algorithms) and prevention strategies in each of the participating countries. The project builds on an experienced, skilled, and cohesive partnership founded through previous collaborations across Europe. All partner institutions are public bodies, representing either public health institutes or universities in their respective countries with skilled and well-equipped laboratories ensuring capable scientific and technical reporting. The Sialon II partnership is rooted more broadly in a further network of local collaborating partners ranging from universities, teaching hospitals, epidemiological centres, gay and/or HIV non-governmental organisations (NGOs) across all participating countries. This extended network has been a key resource for the project ensuring on the one hand, relevant specialisation in HIV/STI research, and on the other, suitability to social, cultural, policy and political contexts in each country as well as actively enabling the participation and views of the target group (MSM) to be taken into account.

In what is to follow, the three related yet distinct components (i.e. formative research; primary data collection; targeted prevention activities) are described in more detail.

### Formative research

During 2011–12, formative research (FR) was carried out in all 13 participating countries. Information was collected via a combination of a dedicated FR questionnaire and focused analysis of secondary data from the European MSM Internet Survey (EMIS) [16, 17]. The FR questionnaires were completed by each project partner from the respective study country in partnership with their local collaborating partner (e.g. NGO). Each questionnaire explored a number of areas such as: the proposed study site; previous experiences with different study methodologies and target groups; data on gay-friendly commercial and non-commercial sites; testing opportunities, regulations, and treatment guidelines for HIV, STIs, HBV, and HCV; contextual factors such as legislation relating to gay issues (e.g. date of homosexuality becoming legal, possibility of gay marriage or officially recognised civil partnerships, protection from discrimination regarding sexual orientation etc.) and stigmatisation. In addition to the contextual information provided by the country partner, EMIS data were also used to further characterise the MSM population in each respective study area. This allowed for compiling a comprehensive ‘picture’ of each study area which described the relevant context, in which the data collection took place, included information such as demographic characteristics, proportions of MSM among the adult population, as well as data such as the degree of ‘out-ness’ reported by MSM in the study area, self-reported internalised homo-negativity, gay-venue attendance, and self-reported HIV and STI history.

During the FR process, gay-friendly commercial (e.g. cafés, bars, discos, sex-clubs, saunas, porn-shops) and gay friendly non-commercial sites (e.g. community centres and cruising areas) were described in detail using a number of indicators such as the estimated number of MSM attending the venue per day during a week, opening hours, special events, logistical aspects for performing sampling, opportunities for sexual contact in the venue, availability of condoms, lubricants and other prevention activities, and so on.

Findings from the FR were presented and discussed during a dedicated study meeting with all network partners present (including collaborating partners such as NGOs). A FR report was compiled and provided to all national study sites in order to better tailor both the planned TLS and RDS surveys, as well as the targeted prevention actions [17].

### Primary data collection

Following, and informed by, the FR phase, a TLS survey (3,661 participants) was planned and implemented during 2013–2014 in nine cities and countries: Belgium (Brussels), Bulgaria (Sofia), Germany (Hamburg), Poland (Warsaw), Portugal (Lisbon), Slovenia (Ljubljana), Spain (Barcelona), Sweden (Stockholm), and the UK (Brighton). In the remaining four partner countries, an RDS survey (1,305 participants) was planned and implemented in Italy (Verona), Lithuania (Vilnius), Romania (Bucharest), and Slovakia (Bratislava) (see Table 1 - Timeline in the Sialon II study by sampling method and city; Table 2 - MSM enrolled in the Sialon II study by sampling method and city). In all cases the following inclusion criteria for MSM participation were used: must have had any kind of sex (oral or anal, penetrative or not) at least once with another man during the previous 12 months; able to provide anonymous informed consent; agree to complete the study questionnaire; agree to provide either an oral fluid sample (for TLS) or blood sample (for RDS). Exclusion criteria were: being younger than 18 years old, and having already participated in the study.

**Table 1:** Timeline in the Sialon II study by sampling method and city

Sampling method	City and Country	Starting month	Ending month
Time-Location Sampling (TLS)	Brussels, Belgium	April 2013	July 2013
	Sofia, Bulgaria	July 2013	September 2013
	Hamburg, Germany	April 2013	August 2013
	Warsaw, Poland	May 2013	September 2013
	Lisbon, Portugal	April 2013	August 2013
	Ljubljana, Slovenia	May 2013	October 2013
	Barcelona, Spain	May 2013	June 2013
	Stockholm, Sweden	May 2013	August 2013
	Brighton, UK	April 2013	June 2013
Respondent-Driven Sampling (RDS)	Verona, Italy	June 2013	June 2014
	Vilnius, Lithuania	June 2013	August 2014
	Bratislava, Slovakia	June 2013	August 2014
	Bucharest, Romania	February 2014	November 2014

**Table 2:** MSM enrolled in the Sialon II study by sampling method and city

Sampling method	City and country	Overall sample
Time-Location Sampling (TLS)	Brussels, Belgium	406
	Sofia, Bulgaria	411
	Hamburg, Germany	408
	Warsaw, Poland	408
	Lisbon, Portugal	409
	Ljubljana, Slovenia	416
	Barcelona, Spain	408
	Stockholm, Sweden	377
	Brighton, UK	418
Respondent-Driven Sampling (RDS)	Verona, Italy	400
	Vilnius, Lithuania	322
	Bratislava, Slovakia	400
	Bucharest, Romania	183
	TOTAL	4.966

Prior to biological sample collection in both TLS and RDS study arms, informed consent was collected for each participant. A self-administered pen-and-paper questionnaire was then administered to all participants to obtain data on: the social and cultural context of respondents; behavioural data on sex practices; risk-reduction strategies such as not having anal intercourse with non-steady partners, condom use, and HIV serostatus disclosure; STI history; self-reported serostatus, and number and type of sexual partners. The questionnaire was designed by the Sialon II network in line with the GARPR indicators. A preliminary version of the questionnaire was piloted in each country with the collaboration of local gay and/or HIV NGOs. Subsequently, a ‘final’ English version of the questionnaire was translated into each of the languages of the participating countries and then back-translated into English for quality control. The same questionnaire was used in both surveys (TLS and RDS): in the TLS version additional items were included focusing on the venues in the given city (for weight calculations), whilst in the RDS survey extra items were used in order to assess the network size of the participants.

In both surveys, in line with the project protocol, a unique identification number (barcode) was used in order to identify each questionnaire and to link the behavioural information with the biological data (e.g. oral fluid). This approach was used in order (i) to guarantee the privacy/anonymity of the participants and (ii) to minimise the potential for any mistakes in linking the different types of information (bio-behavioural in both TLS and RDS survey.

### Time-location sampling

TLS has been used successfully in previous studies and has demonstrated to be an effective and reliable method for gathering both behavioural and biological data in ‘hard-to-reach’ populations [14, 15, 18–20]. Based on assumption of an HIV prevalence in the study population of at most 15 % based on results from the previous Sialon project, a precision of 5 %, a significance level of 95 % and a design effect of 2, a random clustered sample size of 392 MSM per city was calculated. Taking into account the possibility of invalid samples, a final target of *n* = 408 per city was planned.

With regards to venues and participants eligibility, any physical public or private locations attended by MSM could be included as venues in the universe (e.g. commercial venues such as cafés, discos/clubs, bars, sex shop, sex cinema, saunas, spas etc. as well as non-commercial venues such as cruising settings and special events). One exception were venues that specifically serve HIV positive members of the priority population, since including these types of venues would artificially increase representation of HIV positive individuals in the final sample. Virtual meeting places such as websites and smartphone apps were not included.

In order to finalise the sampling frame, once the initial list of venues or initial universe of venues was elaborated based on the findings of the formative research, MSM venues and venue-day-time (VDT) units were identified and two sampling frames constructed [19]. The first sampling frame (or venues sampling frame) comprised a list of venues that met the attendance requirements and were willing to participate (eligible venues). The second sampling frame (VDT sampling frame) comprised a list of venue-specific sampling periods of four hours each (VDT). In constructing this second frame, for each venue and for each day of the week, the VDT units were determined according to two key criteria: i) logistical feasibility and safety for data collectors; and ii) VDTs were expected to yield at least 10 eligible MSM. Consequently, at this stage some venues identified previously as being potential data collection sites were excluded due to various reasons such as low levels of attendance of eligible MSM participants, specific attendance patterns, and/or operational barriers or lack of permission by the venue owners/manager.

Where possible, in order to verify characteristics of potentially eligible VDTs, project partners and/or their collaborating partners (e.g. NGOs) interviewed the respective venue owners. This process also allowed (where necessary) the counting of MSM attending the VDT by use of a ‘clicker’ (Type I Enumeration) [21] assuming that most attendees would be eligible. In order to discern venue specific sampling periods, standardised enumerations were conducted of the MSM attending venues during possible high attendance day-time periods. The result of this process was the creation of the final VDT sampling frame. As data collection proceeded, these sampling frames were then updated on a monthly basis (e.g. where new venues may have opened, others closed, or the VDT proved to be unproductive).

Following completion of the final sampling frame, a three-stage sampling plan to select venues, VDTs, and participants was used. Based on the monthly sampling frames, a set of venues equal to the number of sampling events planned for the upcoming month were randomly selected (stage 1 of randomisation). Selected venues were then sorted in ascending order of VDTs beginning with the venue with the least number of VDTs (which is less flexible for scheduling on the calendar); one VDT was selected from this venue (stage 2 of randomisation). The VDT selected was scheduled on the calendar by choosing any available day on which the VDT falls. After this sampling event was scheduled, a VDT was selected randomly for the next venue. The process was repeated for each venue selected in first stage of randomisation. Stage 1 and 2 of randomisation were carried out in two independent steps because if they had combined in only one step, the venues with many VDTs would have been more likely to be selected than those with fewer VDTs.

In addition to venues, each country could also select purposefully up to a maximum of three events to schedule into the sampling calendar (infrequent events to attract members of the target population e.g. gay pride), and thus were excluded from the sampling frame. As the sampling calendar was filled, if the selected VDT could not be scheduled because there were no days available in any week of the month in question, a new VDT was selected randomly from the remaining VDTs of that venue. If no VDT from that venue could be scheduled in any week of the month, then another venue was selected randomly from the set of venues not chosen in stage 1, until the sampling-event was filled. If there was no choice, overlapping VDTs were scheduled.

More VDTs than needed were scheduled as alternatives VDTs. When data collectors did not achieve the sample size in the primary venue they could go to an alternative VDT until they had reached the sample size. Alternatives VDTs were scheduled in different days and/or timetable. They also were chosen randomly to minimise selection bias. If an event was cancelled before their occurrence for some unforeseen reasons it was rescheduled, that means to change VDT in another week of the month, the same local, day and period but in another week (alternates venues were not used to replace cancelled sampling events).

Data collection was planned from between two to six months during 2013–2014 depending on the country in question. The decision of exactly when to commence was based on negotiations between the project partner and collaborating partner and informed by the findings from the FR. In the final stage three of the randomisation, eligible men were sampled at selected venues in accordance with the monthly calendar with the aim to collect data from eight men.

### Respondent-driven sampling

RDS [22] was used to recruit representative samples of MSM linked to the gay community in each selected city (Verona, Vilnius, Bucharest, and Bratislava). This specific approach has been used in previous studies [23–25] targeting different sub-groups or populations (e.g.: MSM, sexual workers, IDUs) and has been shown to be a reliable method for gathering both behavioural and biological data in hidden or ‘hard-to-reach’ populations.

As with TLS, formative research was conducted prior to data collection in collaboration with local gay NGOs in each participating city in order to properly identify potential interview sites, incentives (amount and type of incentive), expected network size in the selected city, as well as characteristics of the network structures. According to the RDS literature, for a sample size of 400 between six to eight seeds should be selected. For the present study, five MSM seeds were selected in collaboration with the local gay NGO (NGO representatives and/or MSM recruited in gay venues were designated as seeds by the project team). A specific training session for seeds was organised in each city, where the project aims and methodology were explained, including confidentiality and other ethical issues arising.

Selected seeds were requested to identify and recruit peers from their social network. For this purpose, each seed was given three’recruitment coupons’, containing a unique identification number (barcode), address of the interview location, as well as the names and contacts of the research team. A dedicated software package was developed by the research team in order to generate and print the recruitment coupons (including the identification barcode). This software was designed specifically for generating and managing barcodes to be read using a barcode reader, in order to avoid any potential mistake in the process of code management.

A primary and a secondary incentive were delivered according to the RDS approach [26]. In order to receive the secondary incentive (an incentive for each recruited peer), each participant was required to recruit a maximum of three eligible peers, and each of the recruited peers was required to take part in the survey.

Trained health professionals were involved in the collection of the biological sample (serum) in each sites, whilst trained project staff was involved in the collection of the behavioural information (questionnaire, additional items on the network size information), as well as in the coupon distribution process.

The screener (project staff) clarified the project goals and methodology, ensuring that each participant met the eligibility requirements. Participants were also provided with a short document containing all the information related to the survey. After signing the consent form, the self-administered behavioural questionnaire was presented to the participant and on completion, pre-test counselling was provided according to local standard procedures. A biological sample was then collected by the project team following the local procedures. All biological samples were labelled with the participant’s identification number and sent to the laboratory for analysis. Participants were provided with the appropriate incentive as well as with three numbered coupons (with a specific code number). Instructions on how to select members of the social network were delivered. Finally, the participant was invited to come back to pick-up the results of the tests using a card reporting his unique code. When results were given, a post-test counselling was offered according to local and WHO standards.

### Laboratory testing

In line with the scientific literature [27–29], the algorithms for laboratory testing were developed taking into account both the international gold-standards for STI testing and the local laboratory procedures in each country, as well as the WHO-STI surveillance guidelines.

### TLS study

HIV antibodies were tested in oral fluid samples using a non-invasive collection method. To collect oral fluid sample, saliva collection devices (ORACOL; Malvern Medical Developments, Worcester, UK) were used. After collection, oral fluid samples were kept between 4 °C and 8 °C and sent to the laboratory for HIV/AIDS in the respective countries no more than 72 hours after collection. Before testing the samples were processed according to the manufacturer’s procedure.

HIV-antibody testing on the oral fluid samples was performed according to the manufacturer’s instructions of GENSCREEN HIV 1/2 version 2, BIO-RAD. All HIV-reactive samples were re-tested with Vironostika HIV Ag/Ab, Biomerieux and double sample volume of oral fluid compared to serum was used for oral fluid testing (the antigen component of the test was not supposed to react if oral fluid samples are used). In the case of an HIV-reactive result in one or both tests, participants who came back for their test results were encouraged during post-test counselling to be re-tested from blood according to the algorithm for RDS study. As a quality control, for each oral fluid sample, a total IgG antibodies ELISA test Human IgG ELISA Kit 1x96, Quantitative/Immunology Consultants Laboratory was used in order to assess the sample suitability for testing. Before testing, each sample was diluted 1/250 by a recovery buffer. Samples below 3.5 titre (cut-off) were excluded from the study as being invalid.

### RDS study

Serum samples collected through the RDS method were tested for HIV, HVB, HCV and Syphilis in each country by EC marked commercial diagnostic kits following usual testing guidelines. Serum samples were tested first with an HIV 4th generation ELISA test. For newly diagnosed individuals, positive results were confirmed from the second sample by a Western Blot. Participants with HIV-positive results were counselled and subsequently referred into local care systems for further management of their HIV status. HIV-positive samples were shipped to a central laboratory under refrigerated conditions for Avidity Index calculation.

In terms of STI testing, for HBV infections all samples were tested in each participating country following usual testing guidelines for HBsAg, anti-HBcAb total and anti-HBsAb testing. According to the tests results and to a detailed interpretation of the Hepatitis parameters, a prompt referral of participants to a reference centre for further management of their status was ensured. For HCV infection all samples were tested in each participating country following usual testing guidelines. Screening testing was performed with ELISA anti-HCV diagnostic kits and HCV reactive samples were confirmed using line/strip immunoassays. Participants with positive results were referred into the local care systems for further evaluation of their status. Finally, for Syphilis, collected serum samples were tested in each participating country using treponemal and non-treponemal tests. Each sample was tested by both RPR and TPHA qualitative test to assist in the interpretation of results. In the case of reactive results RPR and/or TPHA quantitative tests were performed. Participants with reactive results were referred into the local care systems for further evaluating the need for treatment.

### Prevention activities

HIV and STI surveillance for both TLS and RDS have been implemented within the broader context of the Sialon II prevention activities. HIV/STI prevention and testing promotion activities are theoretically grounded in two theoretical concepts, i.e. the Minority Stress Model [30] and the Information-Motivation-Behavioural skills model (IMB) [31]. While the former multilevel model, which predicts health outcomes among minority groups such as MSM has contextual relevance, the latter targets individual, cognitive and motivational factors. The development of the prevention activities has also been informed by the formative research activities (see above). In order to ensure comparability across data collection sites, the Sialon II prevention protocols describe the different prevention and training activities for field implementation including: (i) prevention packages (comprising condoms, lubricants, and leaflets with specifically tailored information) which have been developed, designed, and distributed to the target group throughout the data collection period, and (ii) interactive peer-to-peer health education activities that took place at the end of the enrolment process (or in case the participant declined to be enrolled). These activities focused on improving knowledge and building motivation for safer-sex among the participants along the theoretical dimensions of the IMB model. On the basis of a series of multiple-choice question cards, participants exchanged information on sexual health, HIV/STI prevention and testing with data collectors/educators or seeds. In addition (or alternatively depending on the situation), HIV/STI prevention information has been offered to participants.

As a further support mechanism, a dedicated section of the Sialon II project website was developed to include HIV/STI prevention information as well as details of local voluntary counselling testing (VCT) services. In each country, project teams consulted local NGOs with regards to appropriate ‘MSM friendly’ VCT sites both in the respective cities and at regional levels. This inventory informed the final selection of VCT services made available to respondents through the prevention package. Moreover, TLS data collectors/educators and RDS-seeds received a specific training session based on the Sialon II prevention manual which included information on HIV/STI prevention and testing, adaptable strategies for different situations and settings to empower participants to adopt healthy behaviours, and the role of meaningful HIV/STI surveillance and prevalence data in prevention activities [17]. For RDS, participants additionally benefited from discussing prevention issues during the pre/post-test counselling sessions at the participating study sites.

### Ethical issues

In order to comply with all ethical and legal obligations, in both TLS and RDS surveys the name of the participant has not been collected. As noted earlier, a unique barcode was attached to the questionnaire and to the biological sample for each individual allowing anonymous linkage between the two kinds of information for later analysis. Finally, the result of the tests was made available to the study participants through the same barcode attached to a card given to individuals during the sample collection.

Participants enrolled in the study were asked to provide informed consent. Individuals were given the possibility to withdraw from the study at any time without explanation. In case of a confirmed positive test, participants were offered a confirmation test on a different biological sample and encouraged to get a referral to local care for further checks and treatment in line both with the international guidelines and local standard pathways of care.

In order to ensure no harm for the respondents enrolled in the Sialon II surveys and to harmonise all the data collection procedures, a specific training manual for data collectors has been developed. The coordinators were also in charge of monitoring the local data collection and coaching the data collectors during activities. On-going monitoring activities were carried out so that any potential difficulty or issue in preserving privacy and confidentiality was supervised.

The Sialon II protocols have been submitted to the relevant ethics committee in each participating city, where official approval was obtained prior to any data collection.

The ethics committee which granted approval were: Instituut voor Tropische Geneeskunde, Antwerp (Belgium); National Centre of Infectious and Parasitic Diseases, Sofia (Bulgaria); Robert Koch Institute Charite, Berlin (Germany); Azienda Ospedaliera Universitaria Integrata – Verona University Hospital (Italy); Vilniaus Regioninis Biomediciciniu Tyrimu Etikos Komitetas Vilnius Medical University (Lithuania); Komisja Bioetyczna Narodowym Instytucie Zdrowia Publicznego, Warsaw (Poland); Conselho de Ética do Instituto de Higiene e Medicina Tropical, Lisbon (Portugal); Institute Matei Bals, Bucharest (Romania); Slovak Medical University, Bratislava (Slovakia); Komisija Republike Slovenije za Medicinsko Etiko, Ljubljana (Slovenia); German Trias i Puyol Hospital, Barcelona (Spain); Regionala etikprovningsnamden i Stockholm (Sweden); Faculty of Health and Social Science Research Ethics and Governance Committee, University of Brighton (UK).

Parallel to this process, the protocols were first submitted to the WHO Research Project Review Panel (RP2) for the technical approval in 2012, whilst in 2013 the Research Ethics Review Committee (WHO ERC) was consulted for a special evaluation of the ethical components. The Sialon II protocols were approved both by RP2 and by WHO-ERC, thus becoming part of a WHO collaborating study in February 2013. As a consequence of this, periodical monitoring reports on the status of the survey implementation have been prepared and submitted to the WHO-ERC committee for review and approval.

### Data analysis and main results

Data analysis will be carried out in line with the sampling method adopted in each study site. Data management will be carried out using DataEntry, R (v3.1.0), STATA (STATA Statistical Software Release 13, College Station, TX: StataCorp LP) and SPSS. In line with the sampling approach adopted, estimates will be weighted. For RDS, the software used for the analysis will be RDS Analyst (www.hpmrg.org), a suite of R commands developed by Mark S. Handcock, Ian E. Fellows, Krista J. Gile (2015 RDS Analyst: Software for the Analysis of Respondent-Driven, Sampling Data, Version 0.52). Data will be analysed as part of the protocol in 2015 and results will be available in 2016.

## Discussion

A second generation surveillance approach to monitoring and controlling the HIV epidemic is a critical tool for state-of-the art public health. However to-date, adoption of the SGSS approach is far from systematic in terms of implementation across EU countries. Consequently, reliable, valid and comparable data on undiagnosed HIV prevalence related to sexual risk behaviour in ‘hard-to-reach’ populations such as MSM are lacking. This deficiency is problematic because it leaves a considerable gap in terms of the ability of European Member States to develop and implement targeted and evidence-based HIV/STI prevention strategies. In order to maximise the benefits of SGSS approaches, European Member States need to harmonise their HIV and STI behavioural surveillance systems through the adoption of a common set of indicators in comparable SGSS studies targeting high-risk populations [5].

This paper describes the protocols of the Sialon II project, the aim of which has been the implementation of a large-scale bio-behavioural survey among MSM in Europe using a SGSS approach, as well as the carrying out of meaningful and targeted HIV/STI prevention. These project protocols offer an important contribution to the epidemiology of HIV/STIs as well as European public health more broadly. Specifically, implementation of the Sialon II protocols, which take into account the most recent GARPR indicators [11], offer a unique opportunity to pilot a large-scale bio-behavioural survey in line with the SGSS approach in a substantial number of EU countries. The Sialon II protocol and findings (that will be available on the project website as well as in additional scientific publications from 2016) are likely to contribute significantly to increasing the comparability of data in EU countries through the use of common indicators and in implementing effective public health strategies and policies in areas of high need. In addition to this, data collected through the former Sialon project and through the Sialon II survey could represent the basis for implementing additional surveys – in line with the SGSS – in order to develop trend analysis to monitor the HIV-STI epidemic over time.

Two key discrete yet linked elements of the protocols appear to be particularly valuable for the implementation of the Sialon II surveillance and prevention programme including: (i) the use of TLS and RDS methodologies to access ‘hard-to-reach’ MSM (sub)populations, and (ii) a project philosophy underpinned by principles of community based participation. In terms of the former, as sex between men is often highly stigmatised in many European countries, MSM can be ‘hard-to-reach’ which in turn, can make HIV/STI surveillance as well as prevention programmes and/or interventions particularly challenging to implement and evaluate.

In terms of limitations, the use of two different sampling strategies could represent a limitation for the study, as it has been shown in other studies that through TLS and RDS methods different segments of the total MSM population can be enrolled and this can result in different sample characteristics, and the differences may persist also after applying weighting corrections to the result estimates: this might limit the results from the Sialon II survey. However, in Sialon II protocols, the deployment of TLS and RDS methods have been particularly beneficial, as they represent the most advanced and available approaches for generating statistically representative samples from ‘hidden’ and most-at-risk populations (in this case MSM).

Finally, the Sialon protocols may provide a useful and feasible model for community-based organisations to conduct decentralised and de-medicalised HIV testing in the future, as such protocols were tested across highly heterogeneous settings. This approach is increasingly seen as an important strategy to increase coverage of HIV testing and to de-stigmatise and normalise HIV testing in the communities, with the aim to contain the HIV epidemic among MSM.

## Conclusions

It is likely that the implementation of the Sialon II protocols will contribute to a better understanding of MSM HIV/STI prevention needs as well as the existence of gaps with respect to existing HIV/STI prevention provision. Moreover, by involving the MSM community in all aspects of the study through both the partnership itself and collaborations with local gay NGOs in the participating countries, as well as working intersectorally with public health institutions and universities, it is possible to increase the capacity of all stakeholders in using innovative sampling methods for collecting bio- behavioural data among MSM in future second generation surveys. The data collected through implementing the protocols may contribute to estimating HIV/STI prevalence and undiagnosed infections in the MSM population, as well as modelling of STI epidemic patterns, increasing comparability of data in EU countries and implementing effective public health strategies and policies in areas of high need.
